# Changes in resting state networks in high school football athletes across a single season

**DOI:** 10.1259/bjr.20220359

**Published:** 2023-01-18

**Authors:** Connor W. Ghiles, Michael D. Clark, Samuel J. Kuzminski, Melissa A. Fraser, Jeffrey R. Petrella, Kevin M. Guskiewicz

**Affiliations:** 1 Wake Forest University School of Medicine, Winston-Salem, United States; 2 Department of Exercise and Sport Science, University of North Carolina, Chapel Hill, United States; 3 Duke University School of Medicine, Durham, United States

## Abstract

**Objective:**

The aim of this pilot cohort study was to examine changes in the organization of resting-state brain networks in high school football athletes and its relationship to exposure to on-field head impacts over the course of a single season.

**Methods:**

Seventeen male high school football players underwent functional magnetic resonance imaging and computerized neurocognitive testing (CNS Vital Signs) before the start of contact practices and again after the conclusion of the season. The players were equipped with helmet accelerometer systems (Head Impact Telemetry System) to record head impacts in practices and games. Graph theory analysis was applied to study intranetwork local efficiency and strength of connectivity within six anatomically defined brain networks.

**Results:**

We observed a significant decrease in the local efficiency (−24.9 ± 51.4%, *r* = 0.7, *p* < 0.01) and strength (−14.5 ± 26.8%, *r* = 0.5, *p* < 0.01) of functional connectivity within the frontal lobe resting-state network and strength within the parietal lobe resting-state network (−7.5 ± 17.3%, *r* = 0.1, *p* < 0.01), as well as a concomitant increase in the local efficiency (+55.0 +/- 59.8%, *r* = 0.5, *p* < 0.01) and strength (+47.4 +/- 47.3%, *r* = 0.5, *p* < 0.01) within the mediotemporal networks. These alterations in network organization were associated with changes in performance on verbal memory (*p* < 0.05) and executive function (*p* < 0.05). We did not observe a significant relationship between the frequency or cumulative magnitude of impacts sustained during the season and neurocognitive or imaging outcomes (*p* > 0.05).

**Conclusion:**

Our findings suggest the efficiency and strength of resting-state networks are altered across a season of high school football, but the association of exposure levels to subconcussive impacts is unclear.

**Advances in knowledge:**

The efficiency of resting-state networks is dynamic in high school football athletes; such changes may be related to impacts sustained during the season, though further study is needed.

## Introduction

American football entails large-volume exposure to subconcussive impacts. At the high school level, the average number of impacts experienced by football athletes ranges from 175 to over 1400 in a season^1,2^ depending on position, coaching style, and participation in games and practices. The average peak linear acceleration of these subconcussive impacts is between 20g and 30gs, though larger impacts do occur.^1^ For example, Schnebel et al found skill players at the collegiate level experience a 98g impact approximately once every 70 impacts and linemen every 125 impacts.^3^ It is as yet unclear what effect, if any, these impacts may have on the physiology of developing brains in high school football athletes.

One straightforward method of assessing network function is using resting-state functional magnetic resonance imaging (rs-fMRI). Rs-fMRI data are acquired over a period of 5–10 min without the burden of an in-scanner cognitive task, which makes acquisition easy-to-implement and clinically translatable. Resting-state networks are defined by characteristic correlations and anticorrelations in regional hemodynamic responses; these correlations are defined as “functional connections.” Recently, several investigators have analyzed whole-brain connectivity in collision and contact sport athletes. Following exposure to subconcussive impacts in a single contact game of rugby, athletes were found to have altered functional connectivity, with increases and decreases in the strength of functional connections among the orbitofrontal, retrosplenial, and cingulate cortices.^4^ Similarly, in the subacute phase of concussion, the default mode network has weakened functional connectivity and a reduced number of connections in the posterior cingulate and lateral parietal cortices and an increased strength and number of connections in the medial prefrontal cortex.^5^

Graph theory is a quantitative method of comparing the structure and organization of rs-fMRI networks by representing brain networks as graphs composed of nodes (brain regions) and edges (functional connections). Graph theory analysis of both structural and functional brain networks has consistently revealed small world topology, where regions which are closer together have a higher probability of being connected while regions which are further away have a lower probability of being connected. Such small-world networks are defined by a high local efficiency (a measure of the interconnectedness of a given node with its neighbors) and a low characteristic path length (the number of connections between any two nodes) with high strength connections found with closer anatomic location. Because longer axonal connections require higher energy usage, it is thought the evolution of brain networks favors minimizing energy costs, making small world organization essential to their function. Graph theory analyses can be used to describe brain network topology and draw a relationship between changes in both function and organization of networks. For example, Nakamura et al used graph theory to examine neural network properties at different time points during recovery from a traumatic brain injury and found alterations in network properties, including efficiency and strength of functional connections.^6^

The purpose of this study was to examine the cumulative effects of subconcussive impacts on the brain at rest over a season of high school football using a graph theoretical approach applied to rs-fMRI data. We hypothesized that the local efficiency and overall strength of resting-state networks would decrease over the season, but we did not have an *a priori* hypothesis as to what specific networks might be affected, given the heterogeneous nature of head impacts. Rather, we chose to look across all large-scale networks, taking an anatomically driven approach in which we examined the brain in terms of regional changes to local subnetwork organization. We hypothesized that the reduction in network local efficiency and strength would correlate to the frequency of impacts, particularly high magnitude impacts (>50g) sustained during the season. We also sought to examine clinical correlates to any observed changes in network characteristics using pre- and post-season computerized neurocognitive testing.

## Methods

### Participants

This longitudinal cohort study investigated the effects of cumulative impacts over the course of the 2013–2014 high school football season. 17 male high school football players (mean age = 16 years, SD = 0.75 years, range = 15–17 years) were recruited from a local high school football team through an informational meeting that included both the players and players’ parents. Because the individuals were all minors, informed consent was obtained by the players’ guardian(s) and assent was obtained from each player. Player demographic information was collected at the start of the study including, age, playing position, years of football experience, and concussion history. The first MRI scan was held at the beginning of the season and prior to the first full-contact practice in the fall (average 4.6 ± 5.0 days before the first contact practice), and the second MRI was conducted at the end of the season (14.5 ± 8.5 days after the final game). Furthermore, individuals who were diagnosed with a concussion were also asked to undergo an MRI scan within 48 h of the injury in addition to both the pre- and post-season scans. All MRI sessions followed the same protocol. Participants were excluded if they presented with any of the common MRI contraindications or exhibited a structural abnormality in the MRI, such as tumors, hematomas, or intraparenchymal hemorrhages.

### Accelerometer data

To measure head impact biomechanics, players were outfitted with the Helmet Impact Telemetry System (HITS) that was worn during all practices and games (43 practices and 12 games in total). All the helmets were from a single manufacturer (Riddell), and all head impacts exceeding 10g of linear acceleration were recorded locally by the device and transmitted to a sideline unit that would localize the impact.

### Neurocognitive assessment

Neurocognitive function was assessed using a computerized neurocognitive test battery (CNS Vital Signs (CNSVS); Morrisville, NC) during the pre- and post-season. This computerized test battery consists of seven different tests: (1) Verbal Memory Test (VBM) and Visual Memory Test (VMT), (2) Finger Tapping Test (FTT), (3) Digit Symbol Substitution (DSS), (4) Stroop Test, (5) Shifting Attention Test (SAT), (6) Continuous Performance Test (CPT), and (7) Non-Verbal Reasoning Test (NVRT). These seven tests are used to generate age-normalized scores for 10 clinical domains: (1) neurocognition Index (overall neurocognitive status), (2) composite memory, (3) verbal memory, (4) visual memory, (5) processing speed, (6) executive function, (7) psychomotor speed, (8) reaction time, (9) complex attention, and (10) cognitive flexibility.

### Magnetic resonance imaging

Imaging was performed using a GE DISCOVERY MR750 3-Tesla scanner with an 8-channel head coil. Resting-state functional MR images were acquired using an axial plane, gradient-echo echo planar imaging sequence with TR = 2200 ms, TE = 26 ms, flip angle = 79°, field of view = 256 x 256 mm^2^, matrix size of 64 × 64 mm^2^, voxel size = 4 x 4 x 4 mm^3^, and slice spacing = 4 mm. 274 consecutive volumes were collected over a 10 min acquisition period, during which subjects were instructed to remain still, keep their eyes closed, and not to think of anything specifically. In addition, a 3D Sagittal T1 SPGR was acquired for anatomical reference (TE = 1.9 ms, TI = 400 ms, flip angle = 11°, voxel size = 0.93 × 0.93 × 1.2 mm^3^).

### Image processing

Image pre-processing was carried out using the FSL/Python Resting State Pipeline which uses a python wrapper for the FMRIB Software Library (FSL). This pipeline was developed to improve consistency of longitudinal analysis of rs-fMRI data.^7^

In brief, functional MRI images were slice-time and motion-corrected and non-brain voxels were removed. The pre-processed functional scans were then registered to the subjects’ T1 scans before being normalized to the MNI 152 template using FSL’s FLIRT. White matter and CSF masks were regressed out of the functional data and the images were then band-pass filtered between 0.001 and 0.08 Hz. Finally, the images were parcellated into 90 cortical/subcortical regions using the automated anatomical labeling atlas.^8^ The average-time series BOLD signal was extracted for each region, and the Pearson cross-correlation coefficients between each region were calculated. The absolute values of the cross-correlations were used to equally weight both correlations and anti-correlations. These absolute values of the cross-correlations were defined as functional connections, or edges, in the subsequent graph analysis.

**Figure 1.**
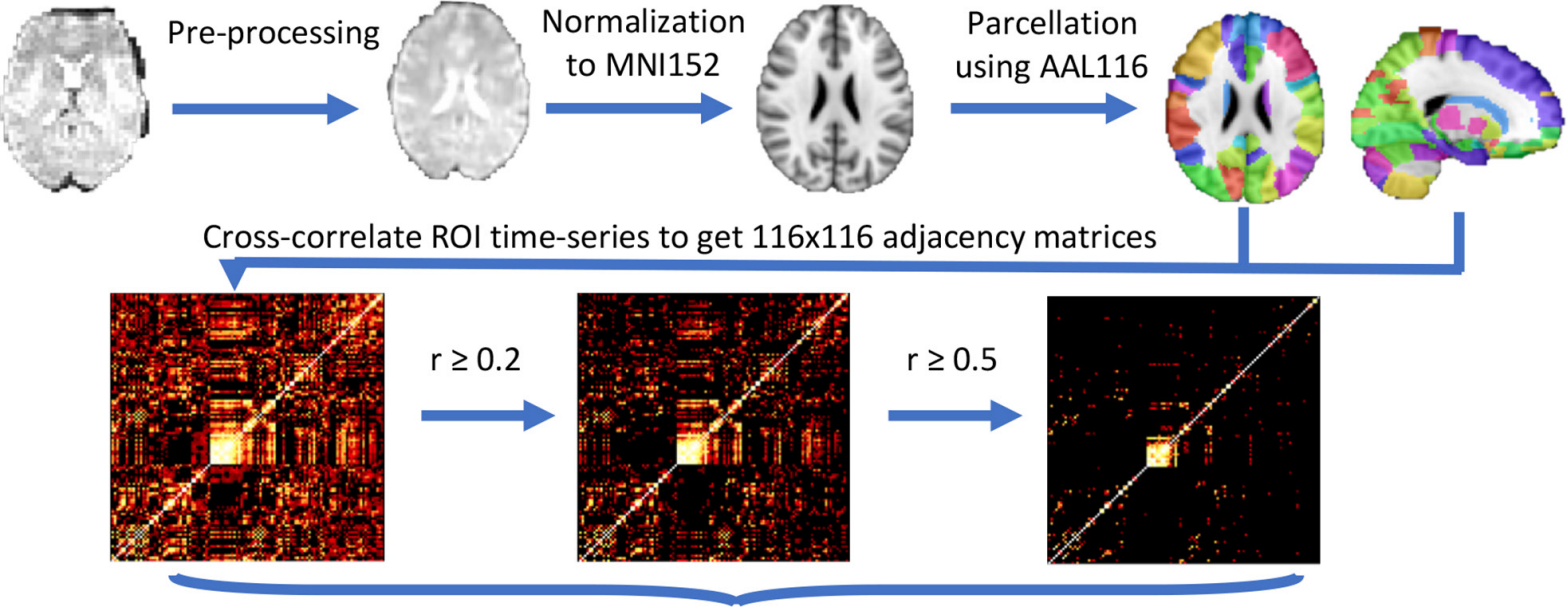
Image processing pipeline showing a representative subject’s MRI through pre-processing, normalization to anatomic reference brain (MNI 125), and parcellation using the AAL116 atlas. Following parcellation, resting-state time series are extracted from each region and cross-correlation (adjacency) matrices are generated for each threshold, r. The graph metrics, local efficiency and strength, are calculated from each adjacency matrix. AAL, automated anatomical labeling.

Mean strength and mean local efficiency were calculated for each subnetwork using the Brain Connectivity Toolbox^9^ in MatLab 2016a (The Mathworks Inc., Natick, MA). We examined both metrics over a range of correlation thresholds from ≥0 to ≥0.9. Two graph theory metrics of interest were derived from the correlation matrices, local efficiency and strength. Local efficiency is a measure of the interconnectedness between nodes; in this study, it is a measure of the correlational structure of resting-state time series data within a brain network. Strength, or nodal strength, is a sum of the connections to a node weighted by the strength of the connection. In this study, strength is the measure of the correlational strength of resting-state time series within a given brain region. The image professing pipeline and derivation of graph theory metrics is diagrammed In Figure 1.

### Statistical analysis

We were primarily interested in three aims: (1) to determine if there were a change in network local efficiency and strength over the course of the season, (2) to assess whether such changes were related to the frequency of impacts experienced over the course of the season, and (3) to examine the relationship between a change in network property and neurocognitive performance.

To address the first aim, we used general linear mixed models in which the graph theory metric is predicted by time (pre- *vs* post-season) with random effects of subject and threshold:

$$LE_{i}=\beta_{0}+\beta_{1}X_{Time}+u_{Subject}+u_{Threshold}+\varepsilon_{i}$$

where LE is local efficiency, β_0_ is the intercept, β_1_ is the coefficient of the fixed effect of time, u_subject_ is the subject-specific random effect, u_threshold_ is the threshold-specific random effect, and ε is the residual error for observation *i*. The random effects for subject and threshold account for the repeated measures design of the study and avoid the choice of an arbitrary threshold in calculating network properties, respectively. A separate but identical model was fitted for strength. Fixed effects estimates were tested against the null hypothesis of no change using *t*-tests with the Satterthwaite approximation to the degrees of freedom (df = 313 for all tests).^9^ Correction for multiple comparisons was performed using the Bonferroni method (*a priori* adjusted *p*-value < 0.05 /N, *N* = number of testing methods, *p* < 0.004). Percent differences from pre- to post-season were calculated by using the *r* threshold with the largest observed difference for the given brain region and graph metric.

To address the second aim regarding the relationship between measures of impact exposure and network properties, we used a similar mixed effects model as above with time replaced by the summary statistics of impact exposure. The summary statistics we used to quantify impact exposure were the frequency of impacts with peak linear acceleration over 10g, frequency of impacts over 50g, the sum of peak linear accelerations above 10g and 50g, the number of years of football exposure, and the number of self-reported prior concussions for each subject. We limited our exploration of this relationship to the networks that showed significant change over the season and restricted the analysis. We used separate models to predict both local efficiency and strength at both time points, as well as the change in these metrics across the season. In modeling the change in local efficiency and strength, we subtracted the pre-season value from the post-season value. Our *a priori* α level for aim 2 was 0.05 for each model.

To address the third aim, we used general linear regression to examine the relationship between changes in network connectivity and CNS-VS scores across all tests and clinical domains. Specifically, we focused on changes in strength and local efficiency in regions that demonstrated significant changes across the season. Initially, the change in both the test scores and clinical domain scores between the pre- and post-season were compared to detect significant changes over these time courses. Pre-season network connectivity metrics (strength and local efficiency) were subtracted from the post-season estimates to create a change score. These were predicted by changes in CNS-VS domain scores for the 10 clinical domain summaries listed above under “Neurocognitive Assessment”. Linear relationships with a *p*-value of <0.05 were considered significant. Because this was an exploratory aim, a correction was not applied to control for multiple comparisons.

## Results

Seventeen varsity high school football players were enrolled in this pilot study (mean age = 16.0±0.8 years). 11 of the participants primarily played offensive positions (65%). One participant sustained a concussion during the study and underwent repeat imaging following his injury. The mean number of head impacts over 10g sustained across the season was 414 (SD = 301 impacts, range = 20–894 impacts) and over 50g was 51 (SD = 49, range = 0–177). See Table 1 for additional demographic and head impact frequency and cumulative impact burden information.

**Table 1.** Demographic information and burden of subconcussive hits in the study participants.

Primary position	Age (Years)	Football experience (Years)	Number of impacts > 10g	Number of impacts > 50g	Cumulative linear sum of impacts > 10g (g)	Cumulative linear sum of impacts > 50g (g)
**OL**	15	5	894	73	24,564.4	4738.3
**DL**	15	4	875	177	31,184.3	12,878.7
**RB**	15	10	860	144	28,593.6	10,496.4
**OL**	16	11	746	52	19,564.8	3352.4
**LB**	16	10	642	77	18,424.9	5397.1
**LB**	17	6	549	63	15,968.8	4442.9
**TE**	16	11	533	61	15,452.3	4123.2
**WR**	16	6	387	69	13,497.6	5757.8
**DB**	16	7	323	21	8164.3	1763.2
**DB**	17	12	283	29	8190.2	1997.8
**DB**	17	4	209	18	5733.8	1363.1
**WR**	16	7	207	15	5017.5	978.2
**QB**	17	7	171	21	4962.7	1599.3
**QB**	16	11	155	40	5634.1	2990.7
**WR**	16	6	99	5	2589	440.2
**WR**	15	9	78	3	1845.5	255.1
**QB**	17	7	20	0	363.6	0
**Mean** (**SD**)	16 (0.7)	7.8 (2.6)	413 (301)	51 (49)	12,338 (9559)	3680 (3536)

### Graph theoretical analysis of resting-state networks

**Figure 2.**
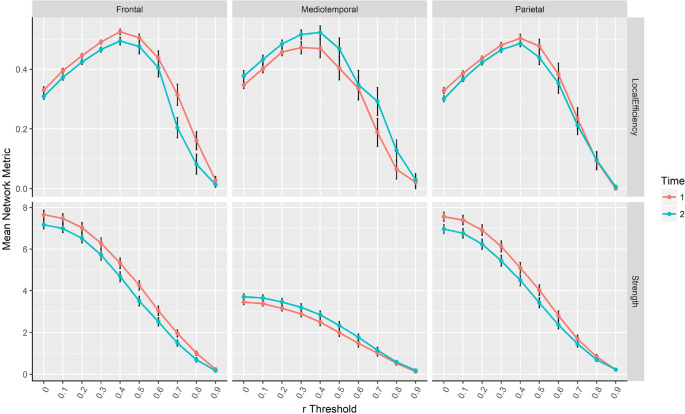
Mean strength (Top) and local efficiency (Bottom) for the frontal, mediotemporal, and parietal networks at each *r* threshold. Time 1 is pre-season and Time 2 is post-season. Error bars show 95% confidence intervals for the mean estimates of strength and local efficiency at each *r* threshold.

For Aim 1, we observed a significant effect of time on the mean local efficiency of the frontal and mediotemporal networks and on the strength of the frontal, mediotemporal, and parietal networks. The frontal network had significantly lower mean local efficiency (decrease of 24.9 ± 51.4%, *r* = 0.7, *p* < 0.01) and strength (decrease of 14.5 ± 26.8%, *r* = 0.5, *p* < 0.01) at the end compared to the beginning of the season, whereas the mediotemporal network had significantly increased local efficiency (increase of 55.0 ± 59.8%, *r* = 0.5, *p* < 0.01) and strength (increase of 47.4 ± 47.3%, *r* = 0.5, *p* < 0.01). The parietal network had a significant decrease in mean strength (decrease of 7.5 ± 17.3%, *r* = 0.1, *p* < 0.01) without a significant change in local efficiency. The mean estimates of strength and local efficiency at each *r* threshold are displayed in Figure 2.

For Aim 2, we observed no significant relationship between impact exposure and local efficiency or strength at either time point (*p* > 0.05). No association was observed for any of the derived summary metrics of head impacts (cumulative linear and rotational accelerations), nor historical indictors of past exposure to head impacts (prior concussion history, year of participation) (*p* > 0.05 for all models). Furthermore, we did not observe a significant relationship between head impact variables and change in either local efficiency or strength (*p* > 0.5 for all models).

For Aim 3, in the frontal lobe, we found a decrease in local efficiency was associated with increased reaction times across the season (F_15_ 6.57, *p* = 0.02). Furthermore, we found an increase in local efficiency correlated with an increase in delayed verbal memory recall (F_15_ 5.34, *p* = 0.04) and an increase in correct CPT response reaction times (F_15_ 6.81, *p* = 0.02). In the mediotemporal lobe, we found an increase in local efficiency correlated with an increase in the simple Stroop test reaction times (F_15_ 7.40, *p* = 0.02). In the parietal lobe, we found an increase in strength was associated with an increase in the number of correct responses in the SAT (F_15_ 10.34, *p* = 0.01). Additionally, we found the increase in strength was associated with a decrease in the following: cognitive flexibility (F_15_ 13.30, *p* < 0.01), executive function (F_15_ 15.78, *p* < 0.01), and complex attention (F_15_ 4.66, *p* = 0.05).

## Discussion

In summary, our study of regional functional connectivity changes in high school football players pre- to post-season demonstrated significantly decreased local efficiency and strength in the frontal and parietal lobes with concomitant increases in both metrics within the mediotemporal lobes. Although the results do not support our initial hypothesis that such changes would correlate with the number of impacts over that season, we did observe associations between the change in network metrics and performance on neurocognitive testing, which suggests that the changes observed in the local resting-state neural networks may be clinically significant. Previous neuropsychological studies have demonstrated correlations between injury to the frontal and parietal lobes and cognitive impairment, including disrupted memory, learning, executive function, and other behavioral disturbances.^10,11^ Local efficiency of resting-state networks has been observed to be lower in patients with a history of traumatic brain injury (TBI).^12^ Subacutely following concussion, local efficiency and strength are believed to be increased within frontal, temporal and thalamic subregions, possibly contributing to post-concussive symptomatology.^13^ While we cannot speculate on the long-term clinical significance of resting-state brain network reorganization, our results are in keeping with previous studies using graph theoretical analysis in the setting of concussive trauma.

The mechanism underlying increased or decreased functional connectivity in different regions observed this study, in possible response to impact or injury, is uncertain, and previous studies have been conflicting in this regard. For example, previous studies using rs-fMRI in mTBI, such as that by Palacios et al, report increased, as opposed to decreased, resting-state functional connectivity in the frontal lobes and parietal lobes.^14^ Key differences between our results and those of Palacios et al are the magnitude, type, and interval since injury. In our study, the second scan was done at the end of the a football season in which only one subject experienced a concussion, while in the latter, the scan occurred roughly 4.1 years after mTBI injury.^14^ Furthermore, the average age of the subjects in our study was 16 years, and in the Palacios et al study, the average age was 27.5 years. Accumulating evidence suggests that resting-state connectivity tends to increase over time after injury, particularly in the default mode network where it is hypothesized that increases in connectivity act as a compensatory mechanism in which loss of structural connectivity, due to damage to axonal connections, is counterbalanced by an increase in functional connectivity of local networks. Murugesn et al observed an increase in the functional connectivity of frontal and posterior default mode network subcomponents across a football season in a group of youth and high school football athletes instrumented with the same impact-recording system used in this study.^15^ Indeed, there is pronounced hyperactivation in the frontoparietal networks in NFL alumni.^16^ It is possible that the younger football players in this study have not experienced subconcussive injury over a sufficient interval necessary to invoke a potential compensatory mechanism.

We have no direct evidence of a causal link between the observed changes in functional connectivity in our cohort and the concomitant changes in neurocognitive performance. Changes in local efficiency of resting-state networks has been observed following TBI and concussive injury.

A limitation to this study was the absence of a control group to validate that the changes observed across the season were indeed because of playing football, as opposed to a non-contact sport. Instead, we used a repeated measures analysis focused only on a single football player group, in which the rs-fMRI at the beginning of the season acted as the baseline, and the mixed effect model was used to quantify a variety of impact exposures for each athlete, including the frequency of hits over a certain force, number of years of prior football experience, and the number of self-reported concussions in the past. An additional limitation was that we confined the analysis to intraregional connectivity rather than evaluating interregional connectivity. Our study focused on the intraregional connectivity differences, which are more stable to physiologic fluctuations at rest and, in the case of task-related fMRI, less affected by changes in task demands compared to changes in long-range connections between areas with dissimilar function.^17^ Graph theory was chosen as the analysis methodology because it requires minimal *a priori* knowledge of the particular networks that may be involved, compared to seed-based and independent component analysis methods that require choice of particular seed regions or resting-state networks.^9^ This methodology was favored because less severe cases of mTBI might be topologically random.^18^

In summary, this pilot study demonstrates significant pre- and post-season changes in intraregional functional connectivity measures in the frontal, parietal, and temporal lobes of high school football players over the course of one season. These regions play an integral role in memory, learning, and other cognitive abilities. Although we failed to demonstrate an association with the number of impacts sustained, we did show an association between change in network connectivity metrics and performance on neurocognitive testing, suggesting that the changes observed in the local resting-state neural networks are clinically significant. Further study is needed to determine whether the reported changes may serve as sensitive markers for detecting subconcussive injury in contact sports and tracking recovery from concussion.
